# Optical Studies of Nanodiamond-Tissue Interaction: Skin Penetration and Localization

**DOI:** 10.3390/ma12223762

**Published:** 2019-11-15

**Authors:** Elena Perevedentseva, Nsrein Ali, Artashes Karmenyan, Ilya Skovorodkin, Renata Prunskaite-Hyyryläinen, Seppo Vainio, Chia-Liang Cheng, Matti Kinnunen

**Affiliations:** 1 Department of Physics, National Dong Hwa University, Hualien 97401, Taiwan; elena@gms.ndhu.edu.tw (E.P.); artashes@gms.ndhu.edu.tw (A.K.); 2 P.N. Lebedev Physics Institute of Rus. Acad. Sci., Moscow 119991, Russia; 3 Faculty of Information Technology and Electrical Engineering, University of Oulu, Oulu 90570, Finland; 4 Faculty of Biochemistry and Molecular Medicine, Biocenter Oulu, University of Oulu, Oulu 90220, Finland; nsrein.ali@oulu.fi (N.A.); ilya.skovorodkin@oulu.fi (I.S.); renata.prunskaite@oulu.fi (R.P.-H.); seppo.vainio@oulu.fi (S.V.); 5 Borealis Biobank of Northern Finland, University of Oulu, Oulu University Hospital, Oulu 90220, Finland

**Keywords:** nanodiamond, bioimaging, skin, fluorescence, autofluorescence, two-photon excitation, FLIM, OCT

## Abstract

In this work, several optical-spectroscopic methods have been used to visualize and investigate the penetration of diamond nanoparticles (NPs) of various sizes (3–150 nm), surface structures and fluorescence properties into the animal skin in vitro. Murine skin samples have been treated with nanodiamond (ND) water suspensions and studied using optical coherence tomography (OCT), confocal and two-photon fluorescence microscopy and fluorescence lifetime imaging (FLIM). An analysis of the optical properties of the used nanodiamonds (NDs) enables the selection of optimal optical methods or their combination for the study of nanodiamond–skin interaction. Among studied NDs, particles of 100 nm in nominal size were shown to be appropriate for multimodal imaging using all three methods. All the applied NDs were able to cross the skin barrier and penetrate the different layers of the epidermis to finally arrive in the hair follicle niches. The results suggest that NDs have the potential for multifunctional applications utilizing multimodal imaging.

## 1. Introduction

The current wide production and use of nanoparticles (NPs) and other nanostructures require a comprehensive analysis of their interactions with living systems using different approaches. Various methods aiming to study NP interactions with biological systems from subcellular structures to the whole organism have been developed [1,2]. NPs can enter the body via different pathways, and one of them is transdermal. This method involves the skin’s response as a physiological barrier, limiting and controlling the interaction with the environment [3]. Recently, NP penetration of biological barriers and their effect have attracted serious interest [4]. Particularly, skin interaction with different NPs of various composition, size, shape, surface properties (for example, TiO_2_, ZnO, quantum dots, silver and gold NPs, etc.) have been observed and analyzed [5,6,7,8,9]. The interaction has been discussed for biological applications in pharmaceutics, medicine, bio-medical studies, imaging, drug delivery and other treatments [10,11,12,13]; and secondly, in terms of nanosafety, particularly for sunscreens containing TiO_2_ or ZnO NPs, or other personal care products [5,7,14] and for industrial NPs [15,16], e.g., C60 in industrial solvents [17].

The use of NPs for local topical drug delivery is currently attracting a great deal of attention. Different models and systems have been suggested and studied experimentally [12,13,18,19,20] or with computational models [21]. However, there are still controversial results on whether or not NPs are capable of penetrating and permeating animal skin and under which conditions. Important questions related to this are under investigation, such as examining the routes and mechanisms of NP penetration required to reach different layers of the skin, and the optimal conditions required. The optimization of methods and approaches is needed.

Understanding the NP interaction with the skin is important for topical, local drug delivery, including nanoparticle-provided delivery of drugs [20,22], such as delivery via the topical application of a nanoparticle-drug system with slow controllable drug release [23]. Among the nanomaterials with demonstrated facilities of controlled drug release, nanodiamonds (NDs) are a promising candidate in the development of multiple applications [24,25]. However, the potential applications of NDs in transdermal drug delivery, as well as other theranostic applications, will depend on understanding the ND interaction with the skin and on the ability to detect and visualize the interaction with the skin.

Recently, due to their physical–chemical properties and variability in their size and structure, NDs have been widely studied and considered as a potentially highly effective material for biomedical applications [25,26]. The optical-spectroscopic properties of NDs allow them to be traced as an imaging agent for different methods of bioimaging. NDs’ fluorescence [26,27,28], Raman scattering [27], and backscattered signal (differential interference and Hoffman modulation ‘space’ contrast microscopy techniques) [28] have been utilized for multimodal imaging and delivery monitoring. The surface properties of NDs allow the functionalization of the particles with molecules of interests for interaction with bio-targets [24,25,29]. Importantly, in anticancer therapy, ND-mediated delivery has been shown to increase the efficiency of drug treatment [30]. In addition, NDs attenuate UV radiation through absorption and scattering and can be considered as a potential sunscreen candidate [31,32].

While the interaction of NDs and ND conjugates with cellular cultures of different types has been studied (including studies of ND penetration through the cellular membrane, distribution in the cytoplasm, problems of endocytosis, clearance with cells in the immune system [33,34]) understanding ND penetration through different tissues, especially tissues such as the skin and the endothelial barrier, is still very much unexplored. On the other hand, ND applications in skin care and skin treatment products have attracted the attention of researchers [35]. Works concerning ND–skin interactions are beginning to be reported. For example, Lim et al. [19] discussed the ability of NDs to penetrate the skin for the first time, but the efficiency and mechanism have not been clarified as of yet.

NP applications are limited because of physiological barriers in the organism manifesting at the cellular and tissue levels [3]. One solution to better understand the NP interaction with those barriers, e.g., the skin, is to combine different imaging methods [36,37,38], allowing the substitution in some cases of invasive histological investigations.

Thus, the goal of the presented work is twofold, and one aim is to examine whether any kind of NDs can cross the skin barrier and penetrate the multiple layers of the epidermis. Based on the properties of the NPs and tissue, the second aim is to optimize the approaches to skin studies to obtain comprehensive information and to carry out a comparative analysis. Additionally, nanomaterials for transdermal penetration allow new approaches for in vivo non-destructive studies. In this report, we combine optical non-invasive imaging methods for both macro- and micro-imaging with different resolutions and imaging depths, including: optical coherence tomography (OCT), fluorescence confocal imaging and fluorescence lifetime imaging (FLIM). OCT is usually applied to clarify relevant tissue morphology and optical/scattering properties [39,40]. This technique can be used to study the effects of some diseases, or other kinds of damage to the tissue [39,40,41]. The interaction between NPs and the skin has also been observed using this technique [42,43]. Fluorescence imaging, in combination with conventional microscopy, provides information on the system’s visible structure and distribution of fluorescent NPs at the cellular level. Fluorescence imaging with two-photon excitation offers multiple advantages, such as low out-of-focus excitation and increasing spatial resolution, which is the safest for biological samples when infrared excitation is used [44]. The beauty of this technique is that it can be combined with measurements of both the fluorescence lifetimes of the NPs and the bio-object’s autofluorescence with FLIM. In addition to the visualization and analysis of the NP distribution, FLIM can be used to study the interaction between NPs and the sample via the simultaneous estimation of the sample conditions by analyzing the lifetimes of endogenous fluorophores and their spatial distribution [18,36,45,46,47].

In the present work, we combine the three methods described above in order to visualize NDs in the skin and to evaluate the applicability of these methods to analyzing the interaction of NDs of different sizes and properties with murine skin in vitro, as well as to estimate their penetration and distribution (in the epidermis, dermis, and hair follicles). Our findings show that NDs can penetrate the skin and can be detected with an appropriate method depending on the ND properties. The obtained results are also discussed from the perspective of further ND applications such as their use as imaging agents (OCT contrast agents, fluorescence markers for confocal fluorescence, two-photon and lifetime imaging) and for drug delivery.

## 2. Materials and Methods 

### 2.1. Nanodiamond Preparation and Characterization

NDs of nominal particles sizes of 100 and 50 nm (High Temperature/High Pressure (HTHP) synthesized) (Kay Diamond Products, Boca-Raton, FL, USA), and detonation NDs (DNDs) with average crystallite sizes of 3–10 nm (Microdiamant AG, Lengwil, Switzerland) were used in this study. The NDs were prepared for the investigation and the characterization was done as previously described [48]. In brief, the NDs were treated with a 3:1 mixture of concentrated H_2_SO_4_ and HNO_3_ at 100 °C for 24 h, to remove surface impurities, non-diamond carbons, and for the carboxylation of the NDs’ surfaces to form a COOH surface group; then, they were separated with centrifugation and washed with bi-distilled water. The pH of the ND water suspensions was adjusted to neutral by adding NaOH or HCl, measured with a Sentron Titan pH-meter (Sentron, Leek, Netherlands).

For the characterization of the ND’s properties, the ND particle size distributions and ζ-potential were estimated using the dynamic light scattering method (DLS) with a Zetasizer Nano ZS (Malvern Instruments, Malvern, UK). The ζ-potential of the carboxylated NDs suspended in water solution was ND −37 ± 3 mV for 100 nm ND, 25.0 ± 1.5 mV for 50 nm ND, and 18.8 ± 2.6 mV at pH 6.50 ± 0.17 for DNDs.

Further, for the application to skin, water suspensions of NDs and a 10× physiological solution (PBS) were mixed to obtain ND suspensions in PBS. The final ND concentration was 1 mg/mL. The same concentration was used for all ND suspensions. The size and shape of the particles, as well as the aggregation, were estimated with the DLS method and with scanning electron microscopy (SEM) imaging using an SEM (JEOL, Tokyo, Japan). The surfaces of the NDs were analyzed using Fourier-transform infrared spectroscopy (ABB Bomem MB154 FTIR spectrometer, Zurich, Switzerland) and the carboxylation was confirmed via observed characteristic lines of C=O (720–1780 cm^−1^) and O-H bending (1620–1640 cm^−1^) of carboxyl groups [49]. The structure of the NDs was analyzed with Raman spectroscopy using an α-SNOM spectrometer (Witec, Ulm, Germany) with a 488 nm wavelength laser excitation (Melles Griot, Rochester, NY, USA). The absorption spectra were measured for ND water suspensions using a Jasco V550 UV/visible spectrophotometer (JASCO International Co., LTD., Tokyo, Japan). The fluorescence spectra of 100 nm NDs has been measured at 488 nm excitation with an α-SNOM spectrometer and Renishaw spectrometer (Renishaw, Wotton-under-Edge, UK) at 532 nm excitation.

The cytotoxicity of the used NDs has been previously estimated to be low by the authors via standard MTT test using a human lung cancer cell line A549 culture [27,50]: the cell viability in treatment with NDs in concentrations up to several tens μg/mL in a cellular growth medium is comparable with the control. To prevent ND aggregation, ultrasound treatment [48] was performed. The ND suspensions were subjected to ultrasound treatment (Transsonic T460 Elma, Labexchange, Burladingen, Germany) for 10 min just before application.

### 2.2. Skin Sample Preparation

The animal care and experimental procedures in this study were carried out in accordance with Finnish national legislation on the use of laboratory animals, the European Convention for the protection of vertebrate animal used for experimental and other scientific purposes (ETS 123), and EU Directive 86/609/EEC. The animal experimentation was also authorized by the Finnish National Animal Experiment Board (ELLA) as compliant with the EU guidelines for animal research and welfare.

The Crl:CD1(ICR) strain for white mice (2 mice), and a black C57BL/6NCrl (8 mice) strain of 6–8 weeks of age were used for the experiments. These two strains were used to determine whether the skin pigmentation would affect the ND detection upon the interaction with skin. The mouse strains were provided by the Laboratory Animal Center of the Oulu University. Both strains are from the Charles River laboratory, in Germany.

The mice were euthanized by cervical dislocation and the back was shaved using a hair clipper. Skin patches were washed in different washes of: betadine 10%, alcohol 70%, and sterile water (2 min each). Then, the patches were immerged in sterile phosphate-buffered saline (PBS) for 1 min. Skin samples were cut into small pieces (10 mm diameter), then distributed in different wells (Cellstar^®^ Tissue culture plates, Greiner Bio-one, Kremsmünster, Austria). A small chamber was installed on the top of each skin sample tightly adjoined to the skin surface so as not to mix the ND samples with the surrounding DMEM medium (4.5 g glucose, Gibco, Invitrogen, Dun Laoghaire, Co Dublin, Ireland) supplemented with 10% of fetal bovine serum (Gibco, Dublin, Ireland) and 1% penicillin–streptomycin (Sigma-Aldrich, St Louis, MO, USA). NDs’ suspensions in PBS (250 µL) were applied into the chambers. As controls, the same volume of PBS or medium were applied. The skin samples with NDs or without were incubated for 24 h at 33 °C and 5% CO_2_.

After incubation, a chamber containing ND suspension or PBS was removed and samples were fixed with 4% paraformaldehyde (PFA). The fixed samples were subjected to further optical-spectroscopic analysis. Between measurements, the samples were kept in the PBS (at 4 °C). After the collection of the necessary OCT data, the samples from the white skin were cleared by Benzyl benzoate and benzyl alcohol mixture (2:1) (Sigma-Aldrich, St Louis, MO, USA), according to the method previously described in [51], and measured with OCT. The samples were divided into two parts: one was used for OCT measurements, and the second was cut into cross-sectional slices to proceed for confocal and FLIM measurements.

### 2.3. Optical-Spectroscopic Analysis

#### 2.3.1. Optical Coherence Tomography 

OCT measurements were taken using a high-speed spectral domain Hyperion OCT imaging system (Thorlabs, Inc., Newton, NJ, USA) with a broadband light source, with a center wavelength of 930 nm, axial resolution of 5.8 µm and lateral resolution of 8 µm (in air). The output optical power of the device was below 5 mW and the axial scanning rate was 110 kHz.

The sample was placed in a Petri dish and immersed in PBS to avoid dehydration (note that PBS to some degree hampered the OCT measurements, but using PBS allowed standardizing the conditions of the experiment).

2D and 3D OCT images were obtained. The 2D images were used for further quantification of the results. The 2D images were measured for 2 s. The images contained up to 1024 × 1024 pixels; 12–40 images for each sample were analyzed and used for calculation of 1D in-depth reflectance profiles (A-scans) by averaging the OCT images in the lateral direction of the selected regions. 3D images were used to illustrate the clearing effect.

To calculate the 1D reflectance profile, 5–6 parts of images without artifact reflection/scattering (from the hair and the solution surface) were selected for every processed image. Origin software was used to transfer the image to a numerical matrix and to select the columns for calculation. In total 100–200 matrix columns (corresponding to individual A-scans) were normalized and then averaged for every OCT image. The results obtained from several images were averaged for each sample. The standard deviation was also calculated. The A-scans were considered individually or averaged for the analysis of peculiarities and identification of common patterns.

#### 2.3.2. Confocal Microscopy 

Confocal fluorescence images were obtained using a scanning confocal microscope TCS SP5 (Leica, Wetzlar, Germany) to observe the distribution of NDs in the skin and to analyze their penetration. A 20× (air) objective lens to see the skin structure, and a 40× (oil immersion) objective lens to observe the NDs and localize them according to the skin features were used.

A 532 nm wavelength excitation was used to detect the ND signal from the NV^−^ centers in the 650–720 nm range. The power in the focal spot was about 0.8 mW. The autofluorescence of tissue also can be observed at this excitation. Its visible intensity was reduced by adjusting the measurement parameters (detector sensitivity, pinhole, etc.). Z-scans were done to analyze the ND distribution in different layers with a thickness up to 0.1 mm. Confocal imaging also allows spectrum collection; this option was used when it was necessary to confirm that the observed signal belonged to the NDs.

#### 2.3.3. Fluorescence Lifetime Imaging (FLIM)

Fluorescence lifetime imaging was used to visualize the NDs in the skin via an analysis of the lifetime distribution. A Ti-sapphire Chameleon Ultra-II (Coherent, Los Angeles, CA, USA) laser was used for the two-photon excitation with a wavelength of 800 nm; a pulse duration of 140 fs; a repetition rate of 80 MHz. The imaging was performed with a 2D scanner (EINST Technology, Singapore) with 3.5 mW of laser input power. The registration was achieved in the spectroscopic range of 450–650 nm with a PicoHarp 300 (PicoQuant Gmbh, Berlin, Germany) single photon counting system and cooled PMT with Olympus an IX 71 microscope; a 40× objective lens was used. The FLIM data were analyzed using the commercially available software package SymPho Time, Version 5.2.4.0 (PicoQuant Gmbh, Berlin, Germany).

Skin layers in the OCT, as well as confocal and FLIM images, were identified by comparison with anatomic skin structure [52,53].

## 3. Results

### 3.1. Analysis of ND Properties for Bio-Imaging Use

The properties of NDs were analyzed in connection with their penetration and a visualization of their distribution in the skin samples.

NDs of three nominal sizes of the particles (3–10 nm, 50 nm and 100 nm) were used and compared. These were denoted in the text in accordance with the specifications sizes as 100 ND, 50 ND and DND, according to the preparation method. It is important to note that ND properties can vary due to their production parameters and subsequent processing. Thus, on one hand, every used ND should be characterized. On the other hand, the variability of properties gives the possibility to select or to prepare NDs with optimal parameters for the current experiment. In general, the size, surface and optical properties of NDs are crucial in determining their interaction with the skin, as well as the possibility of detection and optimal detection method.

**Figure 1:**
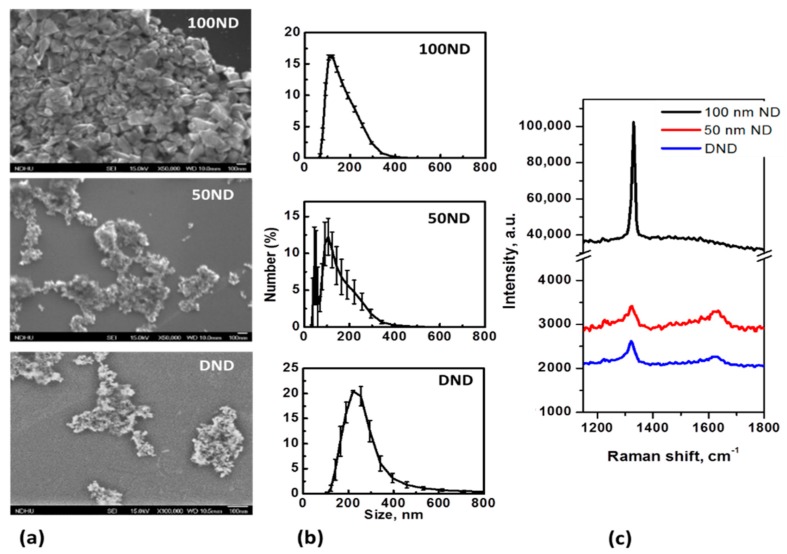
Nanodiamond (ND) characterization: (**a**) SEM images of NDs dried on an Si substrate; (**b**) size distributions in a water suspension and (**c**) Raman spectra of NDs (on Si substrate).

NDs are, in general, polycrystalline particles with a variable non-uniform shape and size. SEM images of the particles positioned on an Si substrate and the size distributions are shown in Figure 1a,b. The average size measured with the DLS method for 100 ND was 150 ± 25 nm, which is close to the nominal size. For 50 ND, the average size was 118 ± 33 nm, but a significant fraction of the particles of a size near 50 nm could also be observed. The size of DND crystallites is usually estimated to be 3–10 nm, but the average size of the particles was measured to be 258 ± 60 nm.

The size distributions of the DNDs show large dispersions and are determined not only by the crystallite sizes and shapes, but rather, by aggregation. Methods to reduce DND aggregation have been developed [54], but it is extremely difficult to prevent the aggregation, especially in biological mediums. In this work, for ND disaggregation ultrasound treatment [48] was performed. Coating with albumin or other convenient macromolecules is considered to be good method to maintain a well-dispersed suspension [55], but in this work, we were interested in analyzing non-coated particles’ interaction with the skin, and considered studying NDs with a modified and functionalized surface as a next step of this research. The measured size distributions of the particles (Figure 1b) are, in general, in agreement with the SEM images (Figure 1a). Separated particles of 100 ND can be seen, while 50 ND and DND appear to be aggregates of small crystallites. Note that usually a fraction of isolated (non-aggregated) NPs, visible in SEM images is also present in the suspensions of 50 ND and DND, but this is not shown by the DLS. The sizes of the crystallites of used NDs and ND structures are also confirmed by the Raman spectra (Figure 1c). The spectra reveal a peak of sp^3^ hybridized carbon, confirming the diamond structure of the particles. For 100 ND, it is at 1332 cm^−1^, corresponding to bulk diamonds, while the values for 50 ND and DND show a widened peak shifted to 1322–1325 cm^−1^, which can be attributed to the phonon confinement effect when the crystallites’ size decreases to a few nm [56].

In a number of previous works, the particle sizes have been shown to be the most important parameter determining NP penetration into the skin [18]. For passive penetration through the trans-epidermal route, mostly particles of sizes in the range from a few nm to 10–20 nm has been considered [38,57,58,59], whereas particles of sizes of a few tens or more nm have been observed to penetrate via the trans-follicular route. The penetration of NPs of sizes of several hundred nm in healthy, intact, human or porcine skin predominantly via hair follicles has been numerically demonstrated. NP penetration and the distribution in the skin depend first of all on the correlation between the NP sizes and the anatomical features of the skin (hair cuticula [36,42]).

For NDs, only one study has been done previously; it showed that DNDs conjugated to a fluorescent marker do not penetrate through human skin in detectable quantities [19]. However, we should take into account that NP penetration into the skin can depend on the skin’s morphological characteristics such as size and thickness, as well as the skin conditions. Additionally, other properties can affect NP penetration into the skin such as: the NP shape, surface charge, composition (including conjugated molecules’ properties, and the hydrodynamic diameter), and physicochemical properties of the solvent. These properties are important in understanding the penetration conditions of NDs into the skin, to optimize the visualization method, and to use NDs as an imaging agent [8,9].

The surface properties of NDs determine not only their tendency to aggregate, but also, to a significant degree, the interaction with the biological target. Negligible cytotoxicity has been demonstrated for the NDs used in this work (data not shown); note, that low or no cytotoxicity has been demonstrated before for different kinds of NDs elsewhere [27,50,55,60]. The NDs’ surface can be functionalized and modified with molecules of interest for interaction for further bio-applications [24,28,61].

In the present work, we use non-modified NDs. Although for applications, the surface of the NDs has to be modified with a bio-active agent, to test non-modified NDs is an important step in understanding the conditions for ND penetration into skin in general, as well as for the optimization of visualization methods of skin interaction with different kinds of NDs and for a discussion of using NDs as an imaging agent in skin studies.

**Figure 2:**
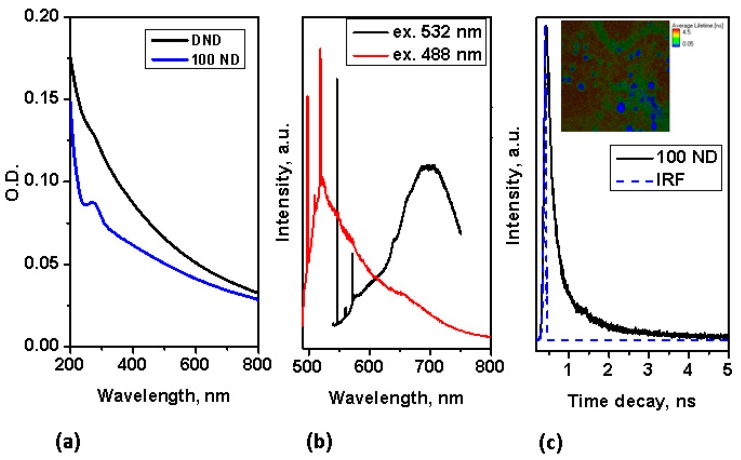
Characterization of spectroscopic properties of NDs: (**a**) absorption spectra of NDs; (**b**) Fluorescence spectra of 100 ND at excitations of 532 nm and 488 nm; (**c**) time decay of 100 ND fluorescence measured at two-photon femtosecond excitation (with a pulse laser at 800 nm); inset-FLIM of 100 ND particles positioned on an Si substrate.

Using NDs for multimodal imaging has been previously demonstrated: the use of NDs as fluorescence and Raman markers has been demonstrated already for a number of bio-systems [27,50,60]. In the absorption spectra, no pronounced peak is observed in the visible range, but strong absorption appears in the UV range, as shown in Figure 2a. Despite this, NDs with a good diamond (sp^3^) structure (such as 100 ND) have an easily detectable fluorescence, which is suitable for fluorescence imaging not only under UV light, but also, under visible and for multiphoton imaging at near IR excitation. The fluorescence of NDs with small-sized crystallites of, high graphite contents (sp^2^ fraction) and a high fraction of surface atoms is low, thus we concentrate here on utilizing the fluorescence properties of 100 nm NDs for multimodal imaging. The fluorescence spectra of 100 ND measured at different excitations in the visible range are presented in Figure 2b. Strong narrow peaks correspond to the Raman scattering of the NDs (near 575 nm at an excitation of 532 nm, and near 519.5 nm at an excitation of 488 nm) and the Si substrate (near 548 nm and 498 nm, accordingly). The origin of ND fluorescence are defects and admixtures in the diamond lattice-color centers [62]. The spectra in Figure 2b show the emission of color centers H^3^ (with the maximum near 500 nm), NV^0^ (with the zero phonon line at 575 nm and the maximum of side band near 600 nm) excited with a 488 nm wavelength and NV^0^ and NV^−^ (with a zero phonon line at 639 nm and the maximum of the side band near 680 nm) excited at 532 nm. It has also been shown that the fluorescence of different centers has different lifetimes [63,64]. Using NDs as a marker for lifetime imaging (such as FLIM) has also been demonstrated [65,66].

In our work, the fluorescence lifetime of 100 ND was measured at two-photon excitation (in the near IR-range), simultaneously utilizing the advantages of two-photon excitation for the imaging of bio-objects [44]. The lifetime decay of 100 ND measured at two-photon excitation is depicted in Figure 2c, in the inset the FLIM of 100 ND particles positioned on an Si surface is shown. This signal was registered in the spectroscopic range of 450–650 nm, including the emission of H^3^, NV^0^, particularly NV^−^ and some other centers [62]. Note, that the two-photon excitation of fluorescence of NV^−^ and NV^0^ [67] and Ni-related 1.4 eV [68] centers has been demonstrated earlier. The measured lifetime of the 100 ND used was short, less than 1 ns (Figure 2c), and allows distinguishing with endogenous fluorophores (the bio-sample autofluorescence) and many exogenous fluorophores, which can be observed or used in bio-imaging.

Additionally, for applications based on fluorescence properties, NDs can be used to increase the contrast for imaging biological systems due to their high refractive index. The refractive index of NDs is estimated to be 2.418 [62], which is significantly higher that the indexes of the many biological tissues. For example, the indexes for substances which constitute the skin are in range 1.3–1.55 [41]. Due to the high refractive index, NDs have already been demonstrated as markers with good backscattering detection for cellular imaging [28]. Considering NDs as a contrast agent for OCT appears to be reasonable and is a subject for further study.

### 3.2. Optical-Spectroscopic Analysis of Skin–ND Interaction

**Figure 3:**
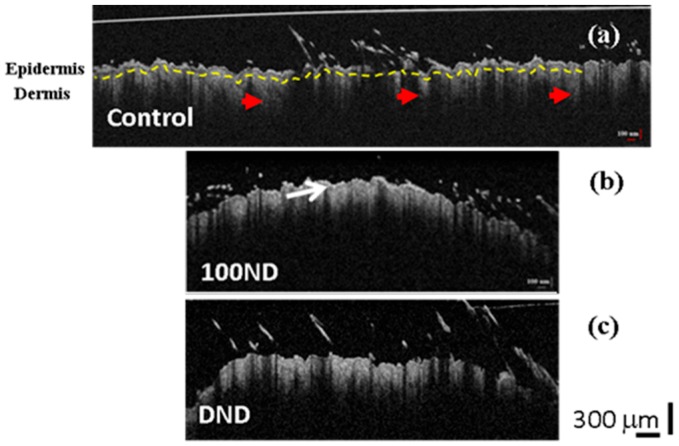
2D OCT images of black skin samples. (**a**) Control samples exhibit normal structure composed of the epidermis, the dermis separated by a basal layer (yellow dotted line), and hair follicles (red arrow heads). Treated skin with (**b**) 100 ND and (**c**) DNDs. The white arrow shows the stratum corneum layer with a higher reflection. The scale bar depicts 300 µm. The experiment was repeated at least three times independently.

In this study, murine skin is used as a skin and tissue model to illustrate the interaction between the skin and the NDs. Note that despite the differences in the structure between murine and human skin, murine skin is recently widely used as model skin tissue [15] for method development and testing in vitro, of in vivo before using a human skin model. Histologically, different layers can be distinguished in murine skin: epidermis, dermis and appendages (such as hair follicles, sebaceous glands and sweat glands) [52,53]. Referring to this structure, OCT 2D images of black skin are presented in Figure 3. Note that there were no visual differences in the OCT images of black or white skin.

The 2D OCT images showed no significant difference between the control skin (Figure 3a) and the treated skin: with 100 ND (Figure 3b) or with DNDs (Figure 3c). In Figure 3, we can observe a clear structure of the skin composed of the epidermis and dermis separated by the basal layer (marked with a yellow dotted line in Figure 3a). However, a thin outer layer with a higher reflectance was observed in both treated skins (Figure 3b (arrow) and Figure 3c), which can be explained by the presence of some stuck NDs in the stratum corneum, together with dead keratinocytes [19]. Because the presence of this additional reflectance in the treated samples, calculations sometimes can be hampered.

**Figure 4:**
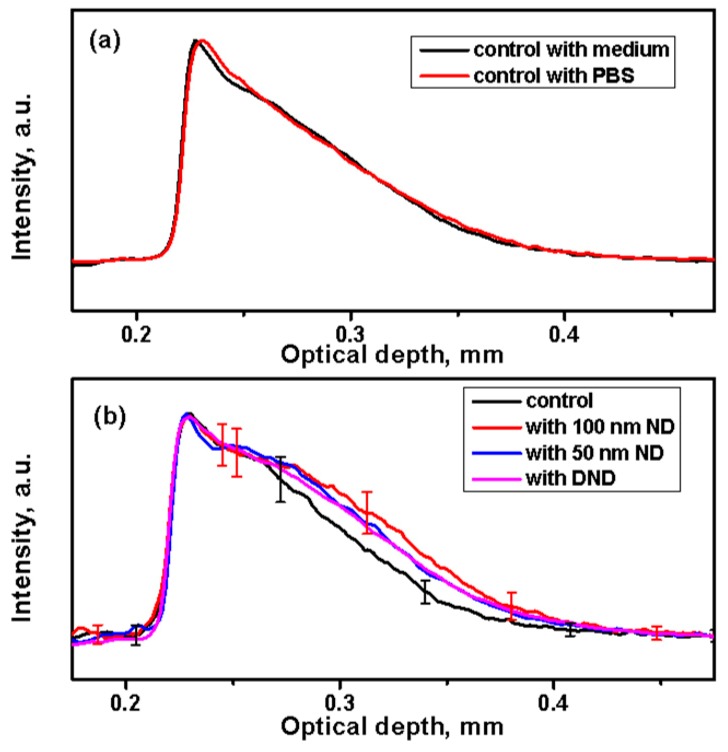
Analysis of A-scan profiles of the backscattering signal of NDs in the skin. (**a**) Averaged A-scan profiles from control samples of black skin after a 24 h incubation in a medium or phosphate-buffered saline (PBS). (**b**) Comparison of averaged A-scans of the control (in PBS) and skin treated with suspensions of NDs for 24 h.

To detect the presence of NDs in the skin, 1D in-depth reflectance profiles (A-scans) were calculated. The profiles obtained by averaging the backscattering signal on the selected regions at fixed points are shown in Figure 4. The A-scan profiles of the control skin from the same strain (black or white) look similar independent of the treatment of the medium or the PBS (Figure 4a).

The A-scan profiles calculated and averaged from the treated black skin with NDs are shown in Figure 4b. They demonstrate higher scattering in all treated samples compared to the control samples. This increased signal can be attributed to scattering from the ND particles dispersed in the skin along its depth. Similar results were obtained from the white skin. Some negligible variations were observed for different mice of the same strain and of different strains. This can be attributed particularly to the skin pigmentation (and to scattering by melanosomes or whole cells containing melanosomes), which varies the optical properties of the skin [69].

Our results from the A-scans are qualitatively comparable to previous reports [43] obtained on the interaction of gold nanoshells with silica core NPs with sizes of about 150 nm and TiO_2_ NPs with a size of 54 nm with rabbit skin in vivo despite significant differences in the experiment design and performance. This data suggests that NPs penetrate the skin and end-up at the junction between the different layers causing the increased contrast.

Despite the fact that the used NDs had different surface electrochemical properties (the ζ-potential of carboxylated 100 nm ND suspended in a water solution was −37 ± 3 mV, while for 50 nm ND it was 25 ± 1.5 mV, and for DNDs it was 18.8 ± 2.6 mV at a pH 6.5 ± 0.17), in this study, we have not observed any effects related to the surface potential. Recent reports indicate that NPs’ surface potential plays a role in their interaction with biological systems in different models (e.g., in protein adsorption, and in vivo biodistribution) [70,71]. On the other hand, the ND environment (in our experiments—medium or PBS) can affect the ND surface. Until now, the mechanisms of these interactions have not been well understood, especially in more complicated systems (tissues, organs). This question deserves more specific consideration.

**Figure 5:**
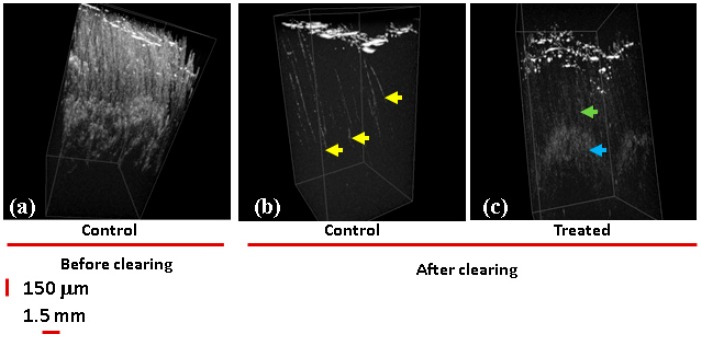
3D OCT images of white skin samples. (**a**) No clearing, control samples incubated with PBS. Both control and samples treated with 100 ND show high levels of scattering on the skin layers. Clearing: (**b**) control; a 3D OCT image reveals scattering areas corresponding to the hair follicles (yellow arrow heads). (**c**) Sample incubated with 100 ND; areas of high scattering are observed distributed in different layers of the epidermis: along the hair shaft (dense, green arrow) and in the hair follicle (cloudy, blue arrow).

The ND penetration was also confirmed by imaging the skin samples after clearing. For clearing biological tissues, osmotically active immersion liquids were applied to the studied samples to reduce the effect of multiple scattering originating from the tissue optical non-uniformities, which limits the depth of OCT imaging [72]. In Figure 5, the 3D OCT images before (Figure 5a) and after clearing (Figure 5b,c) are compared. In Figure 5a,b, control samples are presented. The image in Figure 5a of a non-cleared sample reveals a high level of scattering on the skin layers, while the image of cleared tissue (Figure 5b) shows scattering predominantly by the skin surface and in the hair follicles (yellow arrows).

In the image of the sample treated with 100 ND (Figure 5c), scattering areas are formed in the skin presumably due to the presence of ND clusters stuck in the epidermis. More clearly, a signal was observed starting from the basal layer (dense, blue arrows) and along the hair follicle structures ending in the dermal niches (cloudy, green arrows). This signal is due to the presence of multiple individual NDs or smaller clusters. Based on this, we can suggest that NDs can accumulate in some skin structures, causing alterations of the optical properties of the tissue and increasing the scattering as well as OCT contrast. Note that the observed ND penetration into the skin is in some contradiction with the single previous report on the ND–skin interaction [19], where NDs were not observed inside the skin. It is reasonable to suggest that ND penetration and the observation of NDs in the skin depend on the conditions of the experiment (e.g., particle properties, time of treatment, etc.) and desirable conditions can be selected. Our results show that NDs can penetrate and can accordingly be considered for imaging and for transcutaneous delivery. Additionally, for specific interaction with different cell types or structures, we can expect that modifying the surface of the NDs will allow controlled distribution, localization, and drug release. Moreover, ND–skin interactions should be considered in the case of using NP-containing materials with the skin; particularly, NP-containing cosmetics. In the future, further nanosafety studies and studies on NP or ND biocompatibility with the skin should be carried out.

NPs in the skin are observed to accumulate in some structures, increasing their scattering and contrast. Using NPs as a contrast agent for skin labeling in OCT studies has been suggested before [43,73], but such labeling certainly requires an understanding of the NP distribution in the skin structures. Combining several imaging methods can provide more comprehensive and complementary information about the ND distribution in the skin and its interaction with tissue forming/anatomical elements. Particularly, for 100 ND with detectable fluorescence, OCT images can be compared with different kinds of fluorescence imaging to get more detailed microscopic information and to determine which skin structures are mostly loaded with NDs.

**Figure 6:**
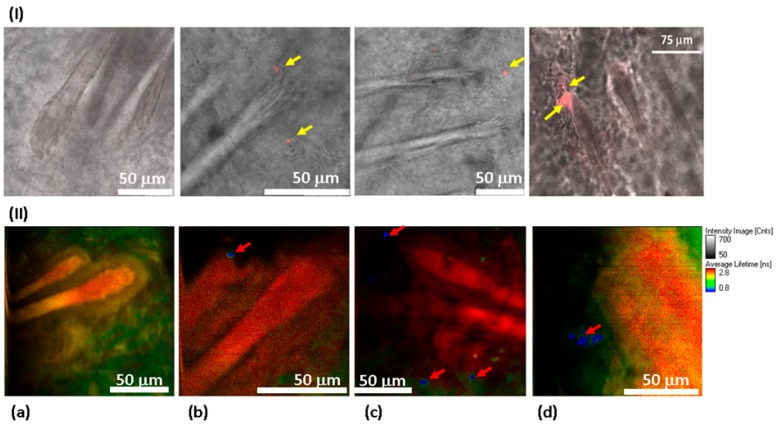
Distribution of 100 ND in the skin using confocal imaging (**I**) and FLIM (**II**). 40× objective lenses (oil immersion for confocal measurements) were used. In confocal imaging (**I**), the fluorescence of 100 NDs was due to excitation at a 532 nm wavelength and was detected in the 650–720 nm range. In FLIM (**II**), a two-photon fluorescence was excited with an 800 nm femtosecond laser, and the signal was detected in the spectral range of 450–650 nm. (**I**) Skin sections observed using a confocal scanning microscope to visualize the fluorescence of 100 ND. No fluorescence signal was detected in the control sample (**I**(**a**)), while the treated sample presents a clear signal corresponding to the presence of 100 ND in different compartments of the hair follicles (shown in red and marked with yellow arrows) (**I**(**b**–**d**)). (**II**) FLIM: (**II**(**a**)) control sample. No fluorescence which could be related to 100 ND is detected. (**II**(**b**–**d**)) 100 ND-treated samples. The short lifetime signal (shown in blue) is visualized, which is characteristic of 100 ND.

To observe the distribution of NDs in the skin via confocal fluorescence imaging, a scan along the z-axis was performed with CW laser excitation in visible range. An example of a z-scan of 100 ND-treated skin can be found in the Supplementary Information, in Figure S2. The confocal skin images (Figure 6I) show the structure of hair follicles. Unlike the control samples (Figure 6I(a)), the treated with 100 nm ND samples (Figure 6I(b–d)) reveal condensate red signals that are localized in different compartments of the hair follicles and in adjacent areas (yellow arrows).

We used FLIM to further confirm and detail these results, (Figure 6II). This allows the visualization of the skin structure in more detail (see Supplementary Material, Figure S1). The lifetime reveals once again the 100 ND distribution in the hair follicles (red arrow, Figure 6II(b,c)). Their localization seems to be in the stem cells’ niche of dermal papilla. Other signals were also detected in the sebaceous gland (the adjacent area of the hair follicle) (white arrows, Figure 6II(c,d)). While no signals attributed to the presence of NDs were observed in the control sample image (Figure 6II(a)), as well as in images of the samples treated with 50 nm ND and DND solutions.

Although the skin samples reveal significant autofluorescence, for both black and white skin, the autofluorescence is low enough in comparison, at least with a large accumulation of NDs. Furthermore, the imaging parameters can be selected to observe only ND signal and to neglect the background.

**Figure 7:**
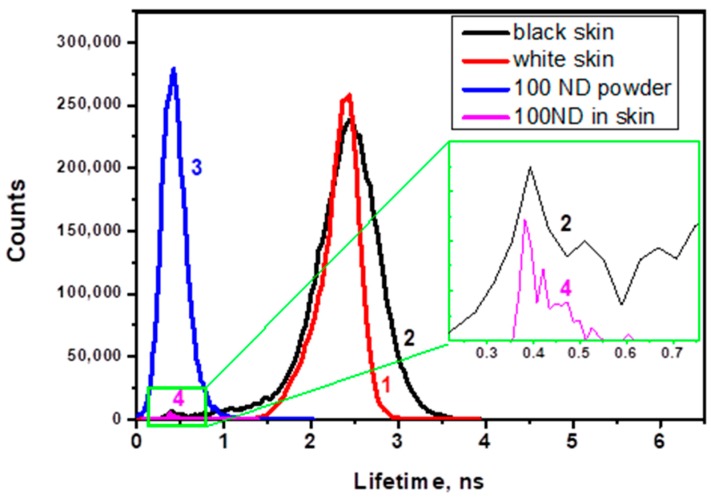
Histograms of the lifetime distribution in images for (**1**) white skin autofluorescence, (**2**) black skin autofluorescence; (**3**) 100 nm ND powder (control); (**4**) observed structure attributed to a cluster of NDs in 100 ND-treated white skin.

Figure 7 shows histograms of the lifetime distribution of the fluorescence lifetime images. The histograms reveal peaks corresponding to the autofluorescence of white and black skin (Figure 7, lines 1 and 2) and to the lifetime of the 100 ND fluorescence (line 4), which is compared with the lifetime measured for ND powder (line 3). The ND lifetime in both cases is much shorter and narrower than for skin autofluorescence.

## 4. Discussion

In this work, the ND signal was found to be predominantly localized in hair follicles, which is in accordance with the literature data, but our data also suggests that NDs can penetrate and become localized in other skin appendages. In Figure 6I(c),II(c–d), 100 nm ND can be observed outside the follicles via ND fluorescence. In agreement, Sirotkina et al. [43] have demonstrated that NPs can penetrate skin appendages. It is suggested [8] that aside from hair follicles, other appendages with openings on the surface, for example, the sebaceous gland or sweat gland pores can provide alternative pathways to cross the stratum corneum.

Based on previous research [18,36,42], we can confirm that several particle properties, e.g., size (results obtained from the SEM image in Figure 1a and from DLS data, Figure 1b) lead to variations in their penetration and distribution. As the NDs can be obtained with a large size range, the behavior of NDs in the skin largely depends on their size.

Our data obtained from OCT and fluorescence measurements after a 100 ND application on the skin are consistent. Confocal fluorescence images (Figure 6I(b–c)) show different localizations of 100 ND in several hair layers (shaft and follicles) or other structures (such as the sebaceous glands) and those adjacent to them. Comparing fluorescence imaging (Figure 6) and 3D OCT images of White skin treated with 100 ND obtained after tissue clearing (Figure 5) generally confirms 100 ND localization: there is an increasing backscattering signal along the hair follicles and a scattering area presumably in between different skin layers (such as the basal layer). Using A-scan profiles, we can only discuss the localization of the NDs in general. An analysis of individual 1D scans indicate that averaging a large number of the 1D in-depth scans undertaken can hide local peculiarities. Additionally, a strong signal of stuck NDs in the stratum corneum and the remaining hair creates visual artifacts that hamper the normalization of A-scans and other calculations (such as averaging).

However, OCT is becoming the main method for detecting the NDs in the skin, treated with 50 ND and DND. The A-scans show an increase of the scattering in the treated samples (Figure 4b), while the fluorescence of these NDs, in the conditions used, is too low for clear microscopic fluorescence imaging with the confirmed separation of the ND signal and skin autofluorescence. However, for 100 ND, both confocal fluorescence imaging and FLIM imaging reveal the ND penetration and localization in the skin via the NDs’ well-detectable fluorescence. A fluorescence lifetime analysis and imaging of skin treated with 100 ND can be realized using two-photon excitation in the infrared range, which is quite convenient for bio-imaging.

In Figure 6 II(a–d), the FLIM of the control skin (Figure 6 II(a)), demonstrating the lifetime of white skin autofluorescence, is compared with 100 ND-treated samples (Figure 6 II(b–d)).

Characteristic lifetimes of skin autofluorescence have been estimated previously with single-photon [74] and multiphoton [75,76] excitations. The lifetimes of skin component autofluorescence vary in a wide range from tenths of picoseconds (ps) to thousands of ps and depend on the skin origin (species), and state (age, diseases, etc.). Due to the above, a numerical comparison of different data is difficult. However, one can say that FLIM allows analyzing the skin structure and probably its state using an autofluorescence lifetime distribution. This reveals structural features, such as hair follicle (specifically stem cells of the dermal papilla niche) and sebaceous glands, to separate the skin layers.

While the FLIM images of non-treated skin demonstrate the autofluorescence lifetime distribution, 100 ND-treated skin shows an additional signal, which can be attributed to the presence of NDs due to its short lifetime and to the localization which is similar to the distribution of ND fluorescence in confocal skin images. Figure 7 shows corresponding histograms of the lifetime distribution of the images. This allows a numerical estimation and comparison of the lifetimes of skin autofluorescence with a 100 ND fluorescence lifetime. The lifetimes of autofluorescence of white and black ND-treated skin samples are comparable (Figure 7, lines 1 and 2), while the lifetime of the 100 ND fluorescence measured for ND powder (line 3) is much shorter and narrower. Line 4 is shown for a signal attributed to a small ND aggregate in an image of ND-treated White skin. The positions of the peak characterizing the lifetime of 100 ND in the ND powder (line 3) and ND aggregated in the skin (lines 2 and 4, inset) coincide, which confirms the attribution of the signal to NDs.

It is worth mentioning that the advantages of using FLIM to study the NP interaction with the skin is that the NP visualization can be combined with studies of the skin state using changes of tissue endogenous fluorophores such as nicotinamide adenine dinucleotide and nicotinamide adenine dinucleotide phosphate NADH/NAD(P)H redox couples [18,77] and for the analysis of the distribution of flavin adenine dinucleotide (FAD), keratin, elastic fibers, etc. [45]. Note also that a lifetime analysis allows the study of the interaction of NDs with certain cellular fluorophores and quenchers and observing the energy transfer between them, which can also be varied with variations of the molecular structure [78,79]. Thus, FLIM and lifetime analyses are good tools to study skin interaction with NPs with detectable fluorescence (such as 100 ND), which can be detected and distinguished in a tissue. The observed NDs penetration into the skin is in particular agreement with works demonstrating the penetration of rigid particles with a size more than a few tens nm into healthy skin via hair follicles, and their distribution inside depending on the NP size [18,36,42].

In general, researchers are interested in the influence on NP penetration routes and efficiency of the NP size, material, mechanical and surface properties (hydrophobicity/lipophilicity or hydrophilicity, charge, surface functionalization by molecular and ionic groups, as well as modification by conjugation with macromolecules) [5,7,8,36,42,80,81,82,83,84]. Among other things, the transfollicular pathway seems to be of special importance for NP penetration into intact healthy skin.

It has been shown that particles of certain sizes [36,42,81] and with lipophilic surfaces [82] can efficiently penetrate into the hair follicles, reaching deeper functional structures, and can be stored there for some time. The lipophilicity of the surface also facilitates the particles’ diffusion into all skin compartments [82].

As for the role of the mechanical properties, for elastic NPs, permeation of the stratum corneum into viable cells has been shown [18]. The authors showed that vesicular particles with sizes of about 100–150 nm are able to penetrate the stratum corneas’ lipid matrix through channel structures forming at the interaction of the vesicles with the skin [85].

Other routes of NP penetration into the skin layers also are considered. While transfolicular routes depend mostly on NP sizes and to a lesser degree, on other NP properties, additional routes can depend strongly on skin conditions. Thus, increased NP penetration into damaged skin in comparison to intact skin has been shown [10,86]. Note, that metallic NPs, particularly gold NPs, could be relevant both for intact and damaged skin [86]. The skin thickness (and accordingly the structure) can also be a significant parameter [84]. It has been shown using rabbit skin with a thinner stratum corneum (10–20 µm in thickness) compared to human or porcine skin that NPs of sizes 54–150 nm and different compositions can penetrate and distribute themselves through the skin. This occurs in hair follicles, and predominantly on the border between skin layers after 30 min of NP treatment [43,73]. In these studies, NPs have been detected via OCT and electron microscopy (TEM) in the epidermis and dermis, and the possibility of penetration via connective tissue inside the cells in intercellular substances is discussed.

Note also that other factors such as mechanical treatment, for example, massaging [46] and hair motion [81,87], and treatments with UV-irradiation [88] or microwaves [89] have been applied and presumably could facilitate and allow control of NP penetration into skin.

Thus, NP penetration into the skin has been considered using highly different approaches and a large amount of data has been obtained, but due to a huge variety of conditions and properties, the data are difficult to systematically analyze, and any new information is useful for a better understanding.

We used very thin mouse skin samples in our experiments. There are only three layers in the adult murine epidermis compared to, generally, six to 10 layers in the human epidermis [90]. We should also note that in our experimental protocol, the hair from the skin was shaved, and this mechanical treatment could affect the stratum corneum layer. These factors could be important to provide conditions for the penetration of NDs of 5–150 nm in size into the skin both via follicles or other open surface pores and by diffusion through the stratum corneum. We also can suggest that 24 h incubation of the skin with an ND suspension is a sufficiently long time for NDs not only to penetrate, but also to re-distribute in the skin structures. Skin appendages are surrounded by a network of capillaries, lymph vessels, nerve endings, dendritic and other cells, which potentially allow materials to diffuse from out of the follicles. Additionally, transcellular and intercellular diffusion has been discussed [9]. Both of these routes involve interaction with cellular membrane components. The possibility of the transcellular pathway for NDs can be indirectly confirmed by a number of previous studies. We have shown that NDs can penetrate into cultured vascular endothelial cells [91]. In addition, the ability of NDs to penetrate other cell lines via clathrin-mediated endocytosis has been demonstrated repeatedly and studied in different experimental conditions [33,60,92]. This interaction can be considered to play a role in the NP redistribution in the skin and its response, at cellular and tissue levels. However, the mechanisms are still unclear and need further investigation.

The demonstrated and discussed photonic properties of NDs are convenient for labeling and multimodal imaging in skin studies and make NDs a promising candidate for development of corresponding ND applications and for the development of methods of analysis of the NP interaction with the skin.

## 5. Conclusions

In summary, the results obtained using three complementary imaging methods show that NDs of different particle sizes and surface structures can penetrate into the skin using the model of murine skin. To detect NDs in the skin and to analyze their localization and interaction with the tissue, the most optimal imaging methods were selected for observing NDs with different optical properties. 100 nm ND was the most appropriate particle to compare the capabilities of OCT, confocal and two-photon fluorescence microscopy and FLIM. Transfollicular localization was observed to be consistent with the previous literature data. However, NDs were also found outside the follicles. This suggests other penetration routes or ND re-distribution.

## Supplementary Materials

The following are available online at https://www.mdpi.com/1996-1944/12/22/3762/s1, Figure S1: An FLIM of skin autofluorescence. The fluorescence is due to a two-photon excitation with an 800 nm femtosecond laser, and the signal was detected in the spectral range of 450–650 nm. The FLIM reveals the hair follicle structure (with stem cells in the dermal papilla niche (red arrow; (a)), sebaceous glandes (cyan arrows; (b)); as well as the different skin layers (c). Analogous structures can be distinguished in bright field images (d) and (e). Figure S2: Example of Z-scans of 100 ND-treated skin (images from 1 to 21 were obtained with a step on the z-axis of 0.65 μm). The signal attributed to NDs is marked by white arrows when seen for the first time.
