# Supplementary material for: Optical Studies of Nanodiamond-Tissue Interaction: Skin Penetration and Localization
Source: Materials (Basel). 2019 Nov 15;12(22):3762. doi: 10.3390/ma12223762 (PMC6888210; doi:10.3390/ma12223762)
Supplement: Supplementary file 1 [file materials-12-03762-s001.pdf]

materials

Optical Studies of Nanodiamond-Tissue Interaction: Skin Penetration and Localization

Elena Perevedentseva [1,2,3], Nsrein Ali [4], Artashes Karmenyan [1,3], Ilya Skovorodkin [4], Renata Prunskaite-Hyyryläinen [4], Seppo Vainio [4,5], Chia-Liang Cheng [1,*] and Matti Kinnunen [3,*]

[1] Department of Physics, National Dong Hwa University, Hualien 97401, Taiwan; elena@gms.ndhu.edu.tw (E.P.); artashes@gms.ndhu.edu.tw (A.K.)
[2] P.N. Lebedev Physics Institute of Rus. Acad. Sci., Moscow 119991, Russia
[3] Faculty of Information Technology and Electrical Engineering, University of Oulu, Oulu 90570, Finland
[4] Faculty of Biochemistry and Molecular Medicine, Biocenter Oulu, University of Oulu, Oulu 90220, Finland; nsrein.ali@oulu.fi (N.A.); ilya.skovorodkin@oulu.fi (I.S.); renata.prunskaite@oulu.fi (R.P.H.); seppo.vainio@oulu.fi (S.V.)
[5] Borealis Biobank of Northern Finland, University of Oulu, Oulu University Hospital, Oulu 90220, Finland
* Correspondence: mattitapanikinnunen@hotmail.com (M.K.); clcheng@gms.ndhu.edu.tw (C.L.C.); Tel.: +358-40-688-6156 (M.K.); Tel.: +886-3-890-3696 (C.L.C.)

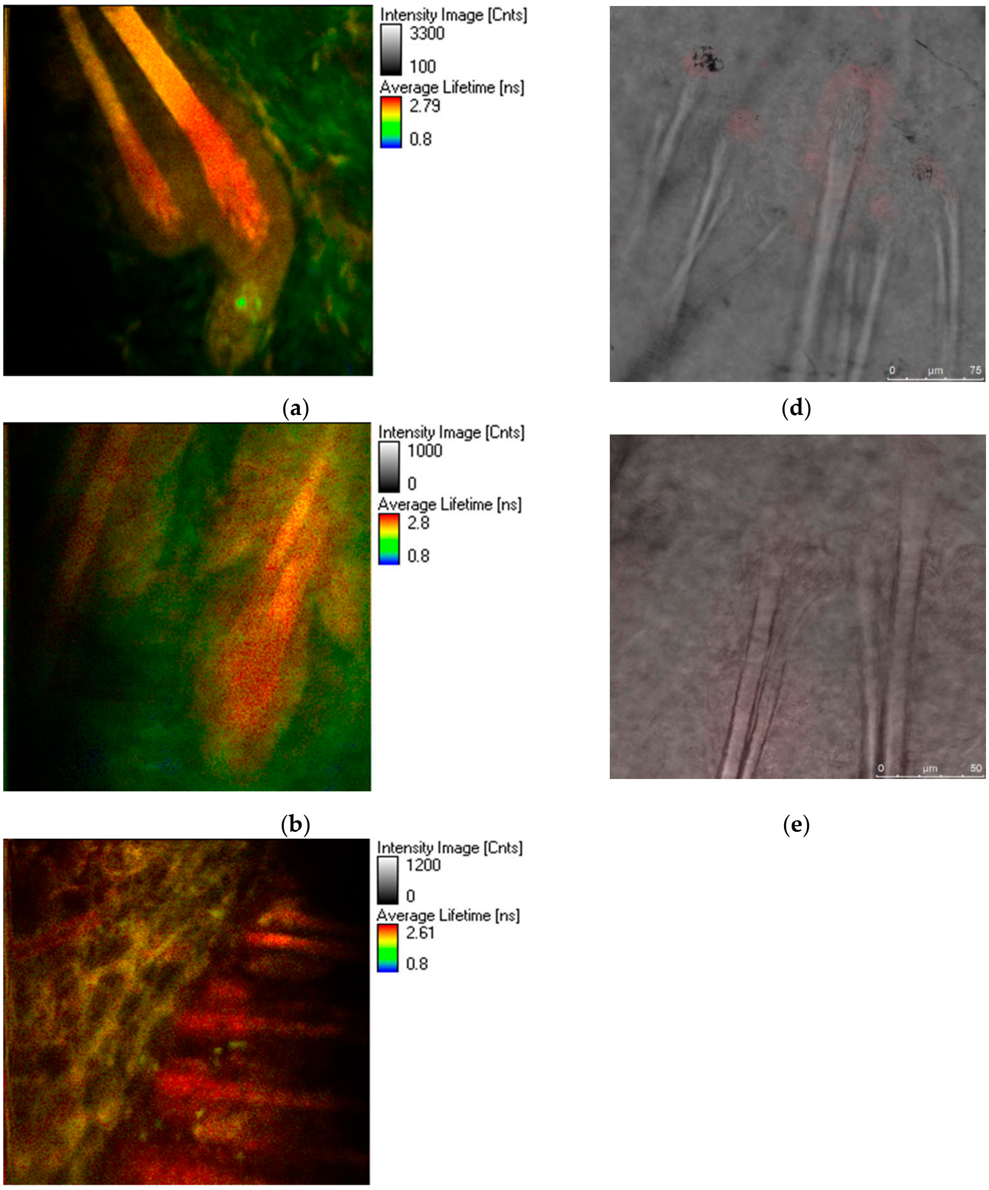

(**a**) (**d**)

(**b**) (**e**)

(**c**)

Figure 1. An FLIM of skin autofluorescence. The fluorescence is due to a two-photon excitation with an 800 nm femtosecond laser, and the signal was detected in the spectral range of 450-650 nm. The FLIM reveals the hair follicle structure (with stem cells in the dermal papilla niche (red arrow; (**a**)), sebaceous glandes (cyan arrows; (**b**)); as well as the different skin layers (**c**). Analogous structures can be distinguished in bright field images (**d**) and (**e**).

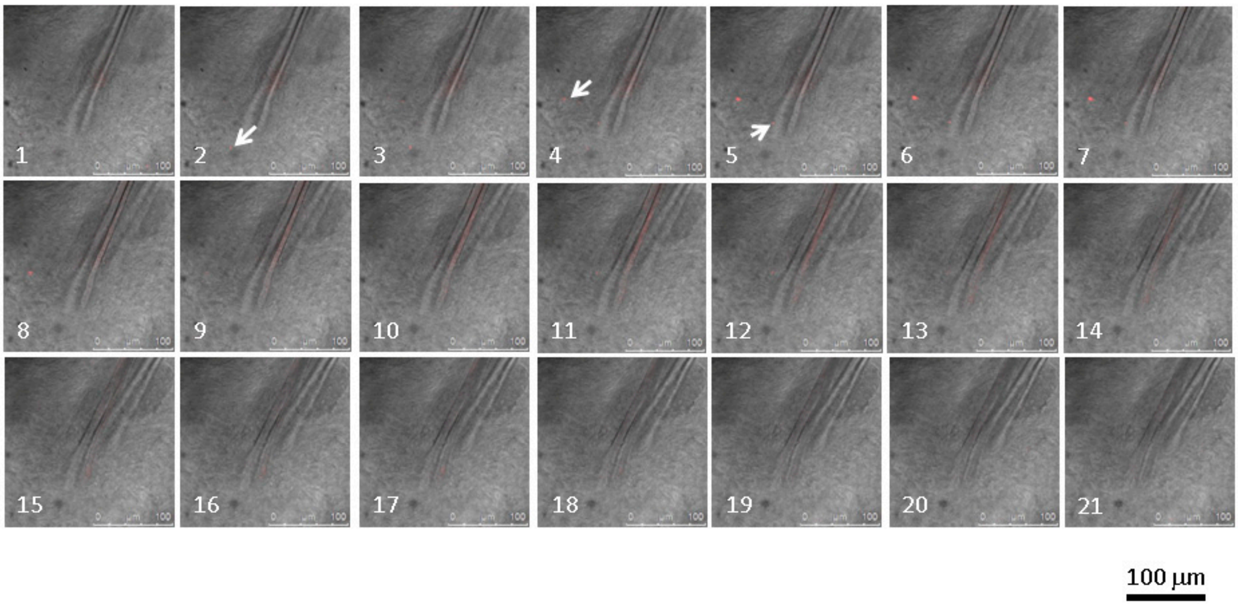

Figure 2. Example of Z-scans of 100ND-treated skin (images from 1 to 21 were obtained with a step on the z-axis of 0.65 µm). The signal attributed to NDs is marked by white arrows when seen for the first time.
